# Calculating the True Costs of Food Service in Long-Term Care: Development of a Costing Methodology

**DOI:** 10.1093/geroni/igaa057.291

**Published:** 2020-12-16

**Authors:** Mikaela Wheeler, Karen Abbey, Sandra Capra

**Affiliations:** 1 University of Queensland, Queensland, Australia; 2 University of Queensland, Brisbane, Queensland, Australia

## Abstract

As population’s age and the need for long term care (LTC) increases, so too does the focus on the costs to provide that care. Providing food, oral nutrition supplements and meals, can be a considerable expense to a home. The objective of this research was to develop a valid foodservice costing tool (FCT), to calculate the real cost of providing foods and meals in LTC. Current costing methodologies are not specific to LTC and do not account for all costs of a foodservice, including staff, procurement and nutrition supplements. An initial tool was developed using the systems approach in conjunction with literature and professional knowledge. This was piloted in real world contexts, using volunteer LTC homes. Four iterations of the tool were completed to assess its feasibility in calculating costs and useability. Managers were interviewed after completing the tool to gather an understanding of how the tool was interpreted and to refine completion. Following feedback, the resulting tool consists of nine sections, measuring both costs incurred in meal production and service as well as analysis of staff workloads. Preliminary results show consistency between homes within Australia, indicating that the true cost is much higher than that reported in the literature to date. The development of a comprehensive, usable tool which captures the total cost of foodservice allows homes to accurately report and understand costs from a systems level. This information can be used to demonstrate cost effectiveness of a foodservice and the potential to justify and plan future system changes.

